# A tumor-associated endothelial signature score model in immunotherapy and prognosis across pan-cancers

**DOI:** 10.3389/fphar.2023.1190660

**Published:** 2023-08-31

**Authors:** Shuzhao Chen, Limei Zhang, Mayan Huang, Yang Liang, Yun Wang

**Affiliations:** ^1^ Department of Hematologic Oncology, State Key Laboratory of Oncology in South China, Collaborative Innovation Center for Cancer Medicine, Sun Yat-sen University Cancer Center, Guangzhou, Guangdong, China; ^2^ Department of Pathology, Sun Yat-sen University Cancer Center, Guangzhou, Guangdong, China

**Keywords:** cancer immunotherapy response, tumor-associated endothelial genes, tumor immunity, biomarker, pan-cancers

## Abstract

**Background:** The tumor-associated endothelial cell (TAE) component plays a vital role in tumor immunity. However, systematic tumor-associated endothelial-related gene assessment models for predicting cancer immunotherapy (CIT) responses and survival across human cancers have not been explored. Herein, we investigated a TAE gene risk model to predict CIT responses and patient survival in a pan-cancer analysis.

**Methods:** We analyzed publicly available datasets of tumor samples with gene expression and clinical information, including gastric cancer, metastatic urothelial cancer, metastatic melanoma, non-small cell lung cancer, primary bladder cancer, and renal cell carcinoma. We further established a binary classification model to predict CIT responses using the least absolute shrinkage and selection operator (LASSO) computational algorithm.

**Results:** The model demonstrated a high predictive accuracy in both training and validation cohorts. The response rate of the high score group to immunotherapy in the training cohort was significantly higher than that of the low score group, with CIT response rates of 51% and 27%, respectively. The survival analysis showed that the prognosis of the high score group was significantly better than that of the low score group (all *p* < 0·001). Tumor-associated endothelial gene signature scores positively correlated with immune checkpoint genes, suggesting that immune checkpoint inhibitors may benefit patients in the high score group. The analysis of TAE scores across 33 human cancers revealed that the TAE model could reflect immune cell infiltration and predict the survival of cancer patients.

**Conclusion:** The TAE signature model could represent a CIT response prediction model with a prognostic value in multiple cancer types.

## Introduction

Although cancer immunotherapy (CIT) has improved outcomes for patients with various types of cancers, only a small fraction of patients experience a durable complete response or derive long-term benefits to CIT (Robert et al., 2014; Ribas and Wolchok, 2018; Robert et al., 2019). Therefore, excavating a new predictive biomarker that can assess the response to CIT for defining a patient’s benefit early is an urgent need. Previous studies have identified some indicators associated with the CIT response, including the glycoprotein VI-mediated platelet activation signaling pathway (Chen S. et al., 2023), NLRP3 inflammasome (Ju et al., 2020), shelterin complex expression (Luo et al., 2021), PD-L1 expression (Patel and Kurzrock, 2015), B cells (Chen et al., 2022), tumor mutational burden (Chan et al., 2018), and eosinophilic count (Moreira et al., 2017). However, accurate biomarkers used for predicting clinical outcomes and CIT responses for cancer patients continue to be largely unexplored.

Immunotherapy has become an indispensable part of advanced gastric cancer (GC), which remains to be malignant with poor prognosis, with a median survival of approximately 12 months (Chen Y. et al., 2023). Many studies have proposed predictive and prognostic biomarkers to immunotherapy agents in gastric cancer. PD-L1 has been proposed as a biomarker for gastric cancer immunotherapy (Ahn and Kim, 2021). Glypican-3 expression in cancer-associated fibroblasts is a critical prognostic biomarker for PD-1 blockage therapy in GC (Li et al., 2023). The T-cell-related gene prognostic index is a potential prognostic indicator used to distinguish the response to immune checkpoint inhibitor (ICI) therapy (Chen J. et al., 2023). The neutrophil-to-lymphocyte ratio is an effective biomarker used for evaluating the prognosis of GC patients who received ICI therapy (Li and Pan, 2023). Unfortunately, only a minority of patients with GC develop durable clinical responses to CIT (Baxter et al., 2021).

Tumor endothelial cells (TECs) are exposed to an extracellular environment that is markedly different from that of the endothelial cells resident in healthy normal tissues (Siemann, 2011). Compared with the vascular system in normal tissues, tumor endothelial markers are highly expressed in the human tumor vascular system and show significant therapeutic potential (Mura et al., 2012). High endothelial venules (HEVs) are specialized blood vessels that are essential for the entry of CD4^+^ and CD8^+^ T-cell lymphocytes into lymph nodes (Girard et al., 2012). Several subtypes of endothelial cells own the characteristics of typical immune cells, including the expression of co-stimulatory and co-inhibitory receptors. Effector T lymphocytes rolled along the inner surface of the vessels or surfaces within the outline of the vessels in the liver sinusoidal and brain microvascular endothelium (Bartholomäus et al., 2009). Through the expression of MHC-I, MHC-II, and a wide array of costimulatory molecules, endothelial cells also act as antigen-presenting cells (APCs) that present antigens to T cells. Moreover, they can act as phagocytes and scavengers to circulate waste macromolecules and participate in efferocytosis (Stamatiades et al., 2016). The interaction between lymphocytes and endothelial cells allows for information exchange that can modify immune responses by the trafficking, activation, and differentiation of lymphocytes. The trafficking of lymphocytes to tumors is critical for CIT. As an active regulator of the immune function, tumor endothelial cells play a potential therapeutic role in immunotherapy in various cancer types. However, whether tumor endothelial gene markers can serve as predictive biomarkers of CIT response needs to be investigated. In this study, we developed a tumor-associated gene signature that predicts immunotherapy across multiple cancer types.

## Materials and methods

### Studies and patient selection

A total of 12 CIT response datasets with gene expression and complete clinical information were included in this study. The datasets obtained by Lauss et al. (2017) (GSE100797), Cho et al. (2020) (GSE126044), Ascierto et al. (2016) (GSE67501), Ulloa-Montoya et al. (2013) (GSE35640), Hugo et al. (2017) (GSE78220), MGH datasets (Auslander et al., 2018) (GSE115821 and GSE168204), and Kim et al. (2010) (GSE19423) were downloaded from the Gene Expression Omnibus (GEO) database (http://www.ncbi.nlm.nih.gov/geo/). Those datasets obtained by Lee et al. (2020) (EGAD00001005738) were downloaded from the European genome–phenome Archive (EGA) database (https://ega-archive.org/access/data-access/), whereas datasets of Gide et al. (2019) (PRJEB23709), Kim et al. (2018) (PRJEB25780), and Van Allen et al. (2015) (phs000452.v2. p1) were obtained from the Tumor Immune Dysfunction and Exclusion (TIDE) database (http://tide.dfci.harvard.edu/) (Weide, 2015). The IMvigor210 cohort was downloaded using the IMvigor210CoreBiologies package in R software (Necchi et al., 2017).

Normalized data obtained from different arrays were normalized using the robust multi-array average (RMA) method (Leek et al., 2012). The batch effect between different arrays was removed using the ComBat function in the sva R package. Patients’ response to treatment was assessed according to the RECIST criteria. The details of immunotherapy response datasets are provided in Supplementary Table S1. The pancan normalized gene-level RNA-seq data and clinical information for 33 TCGA cohorts were downloaded from the UCSC Xena dataset (https://xenabrowser.net/) using the UCSCXenaTools package in R software (Shixiang and Liu, 2019). After applying the data filter criteria, over 10,000 clinically annotated cancer samples with survival information were available for further analyses. The *Homo sapiens* tumor-associated endothelial gene set was defined based on the Gene Ontology (GO) dataset (https://www.informatics.jax.org/vocab/gene_ontology/GO:0003158) and Bagaev et al. (2021) study, including 131 tumor-associated endothelial genes. Furthermore, expressed genes in the transcriptome sequencing data were intersected with 131 tumor-associated endothelial genes to acquire the candidate genes (Supplementary Table S2).

### Construction and validation of the tumor-associated endothelial gene signature

The least absolute shrinkage and selection operator (LASSO) binomial regression model was used to select the best predictive variables, by eliminating parameters with a coefficient of 0 (Tibshirani, 1996). The penalty parameter was estimated by 10-fold cross-validation in the training dataset at 1 standard error beyond the minimum partial likelihood deviance. Using the sample function in R software, a total of 784 patients who underwent CIT were also randomly allocated: 70% were selected as the total training cohort and 30% were selected as the validation cohort. The tumor-associated endothelial signatures were built from the training cohort and validated on the validation cohort. Patients with metastatic melanoma were further divided into on-treatment and pre-treatment cohorts to verify the predictive power of the tumor endothelial signature. Data from patients with metastatic urothelial cancer were subjected to multivariate regression analysis to determine the tumor-associated endothelial signature with independent prognostic significances for survival.

### Immunity analysis

We used the ESTIMATE, EPIC, MCPcounter, Immune AI, CIBERSORT, xCell, TIMER, and single-sample GSEA (ssGSEA) algorithms to evaluate cellular components or cell immune responses between the high- and low-risk groups based on tumor-associated endothelial signatures (Yoshihara et al., 2013; Newman et al., 2015; Becht et al., 2016; Li et al., 2017; Miao et al., 2020; Racle and Gfeller, 2020; Yi et al., 2020). Thorsson et al.'s (2019) data and the TIMER database are used to compare the infiltration of effector cells in the CIT cohort. The signatures of the immune function, immune type, and immune checkpoint were retrieved from previous studies (Ju et al., 2020; Bagaev et al., 2021) and are shown in Supplementary Table S3.

### Gene set enrichment analysis

Using the fgsea package, gene set enrichment analysis (GSEA) was conducted to explore the potential pathways between the low-risk score group and high-risk score group of each cancer type (Yu et al., 2012). The permutations *p*-value was 10,000. The Hallmark gene set “h.all.v7.2.symbols.gmt” and Reactome gene set “c2.cp.reactome.v7.2.symbols.gmt” were downloaded from the Molecular Signatures Database (http://software.broadinstitute.org/gsea/msigdb/index.jsp). *p*-values smaller than 0.05 were defined to be statistically significant.

### Statistical analysis

The area under receiver operator characteristic curve (AUC) was used to assess the prediction accuracy, and the AUC was derived by the R package ROCR (Sing et al., 2005). The Spearman correlation test was used to evaluate the correlation between immune checkpoint signatures and tumor-associated endothelial signatures scores. The coxph function in R was used for univariate and multivariate Cox regression analyses (Therneau and Grambsch, 2013). The survival curves were estimated according to the Kaplan–Meier method and compared using the log-rank test. Meta-analysis of the tumor-associated endothelial signatures was performed using the metafor R package, and hazard ratios with 95% confidence intervals for 33 cancer types were calculated with a random effects model (Viechtbauer, 2010). *p*-values less than 0 05 were considered statistically significant in this study. All statistical analyses were carried out using R software version 4.1.0 (https://www.r-project.org/) and GraphPad Prism software (https://www.graphpad.com/scientific-software/prism/).

## Results

### Patient’s characteristics

We performed this analysis using 12 published datasets with RNA-seq data and clinical response information available for six cancer types (gastric cancer, metastatic urothelial cancer, metastatic melanoma, non-small cell lung cancer, primary bladder cancer, and renal cell carcinoma). These samples were treated with ACT, MAGE-A3, BCG immunotherapy, and ICB therapy, including anti-PD-1, anti-PD-L1, anti-CTLA-4 monotherapy, and a combination of any two. A total of 784 subjects with CIT response information were eligible for downstream analysis. RNA data obtained from the 784 subjects were batch-corrected. We further randomly partitioned subjects into two subsets (70% as the training cohort and 30% as the validation cohort).

### Construction of the tumor-associated endothelial signature

**FIGURE 1:**
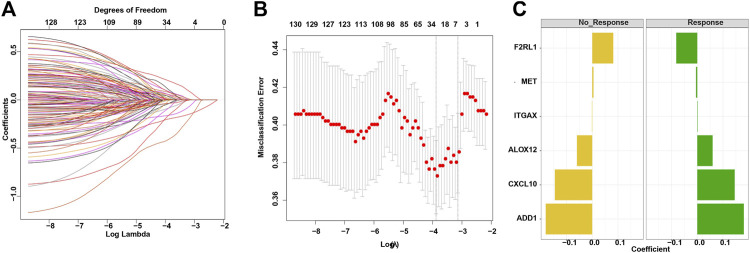
Construction of the TAE score model for a cancer immunotherapy cohort. **(A)** LASSO coefficient profiles of 133 candidate genes. **(B)** Partial likelihood deviance of different numbers of variables revealed by the LASSO regression binary model. Each data point corresponds to the mean of the independent experiments, and error bars denote the standard deviation. **(C)** Coefficient values for each of the eight selected genes. A positive weighting coefficient indicates that the increased expression contributes to the high value for the TAE value.

The LASSO‐penalized binomial regression was used to select the most useful predictive genes from the 131 candidate tumor-associated endothelial genes (Supplementary Table S2). A penalized maximum likelihood estimator was used with 1,000 bootstrap replicates. The optimal weighting coefficients were identified by the regularization parameter lambda via the 1-SE criteria (Figures 1A, B). Regression coefficients were obtained by LASSO-weighted analysis, and a risk score formula was constructed for patients with a responder. Finally, the risk score was calculated by the following formula (Figure 1C): tumor-associated endothelial signature score = 0.180855332*ADD1 + 0.059530341*ALOX12 + 0.145361104 *CXCL10 - 0.081730252*F2RL1 + 0.001225741*ITGAX − 0.004914148*MET.

### Evaluation of the TAE score

**FIGURE 2:**
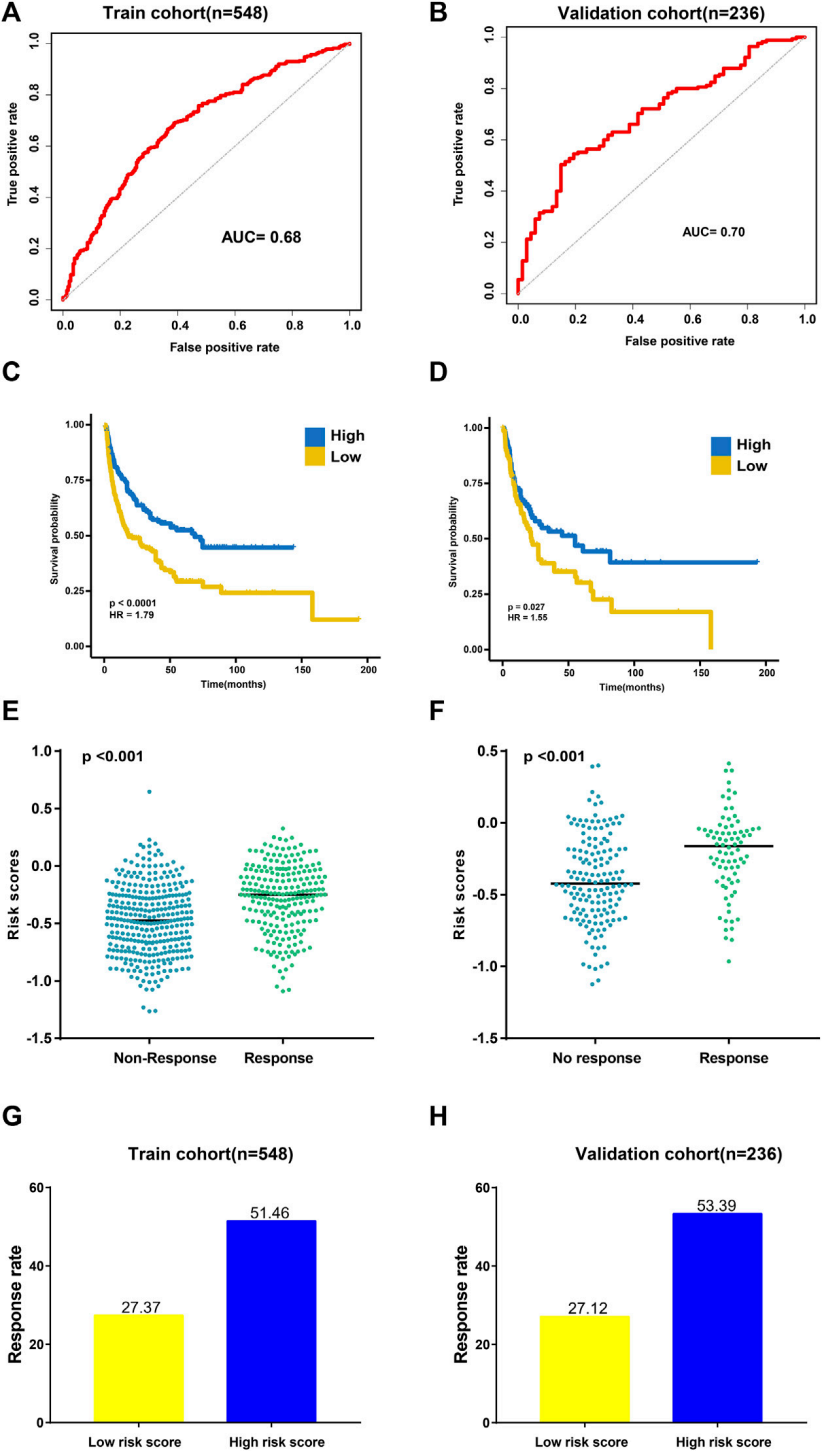
Validation of the TAE risk score model. **(A,B)** Sensitivity and specificity of the TAE score model were assessed in each dataset by time-dependent ROCR analysis. **(C,D)** Kaplan–Meier curves for overall survival. Overall survival by the risk score in the training **(C)** and validation cohorts **(D)** **(E,F)** Distribution of the TAE score among samples grouped by their response to immunotherapy in the training **(E)** and validation cohorts **(F)** **(G,H)**. Response rate to immunotherapy in low- and high score groups stratified by TAE score in the training **(G)** and validation cohorts **(H)**.

To evaluate the performance of TAE classifiers, we used the ROCR package to generated AUC values for determining their potential for differentiating the corresponding responders (R) from the non-responders (NR). The AUC was 0.68 in the training cohort and 0.70 in the validation cohort, which indicated a potential role for TAE signature scores as tools for predicting the response to CIT (Figures 2A, B).

Then, subjects in training and validation cohorts were divided into high- and low score groups, respectively, using the optimal cut-off value of the TAE risk score. In the high score group, patients had a significantly longer OS time than that in the low score group (*p* < 0.05) in the training cohort (Figure 2C). In the validation cohort, the high score group had a longer OS time (*p* < 0.05) as well (Figure 2D). We found that the patients who responded to CIT had significantly higher TAE scores than those with no response in training and validation cohorts (Figures 2E, F). Furthermore, a higher CIT response rate was observed in the high score group compared with patients in the low-score group (Figures 2G, H), which implied that patients in the high score group can benefit from CIT.

Previous studies indicated that on-treatment tumor samples can reliably predict patients’ endocrine therapy responses compared to pre-treatment samples in breast cancer (Turnbull et al., 2015; Bownes et al., 2019), which indicates that we should not only attempt to identify and evaluate the signatures from pre-treatment patients but also attempt to identify and evaluate the signatures in patients who have just started therapy. Considering that only metastatic melanoma patients include pre-treatment and on-treatment tumor specimens, we performed additional tests on them. Interestingly, we found that AUC values derived from on-treatment tumor specimens were significantly higher than those derived from pre-treatment samples (on-treatment AUC: 0.78 vs. pre-treatment AUC: 0.64; Supplementary Figures S1A, B). Patients with high TAE signature scores had a significantly longer OS time than low TAE signature scores in the pre-treatment and on-treatment cohorts, respectively (All *p* < 0.05, Supplementary Figures S1C, D).

Among the CIT cohort, the subgroup of 235 metastatic urothelial cancer patients included complete clinical characteristics, so we further performed analysis on them. The AUC for predicting the response to CIT in metastatic urothelial cancer patients by the risk scores was 0.70 (Supplementary Figure S2A). Patients with high scores exhibited significantly good overall survival (OS) than those with low scores (*p* < 0.001, Supplementary Figure S1B). An independent prognostic analysis showed that the risk score of the TAE prognostic signature was significantly correlated with the survival of metastatic urothelial patients, with HR 0·195 (*p* < 0.001, Supplementary Figure S2C). Furthermore, we used TIDE database data to verify the prediction performance of the prediction model (Fu et al., 2020). The AUCs of TAEs used to predict the response in metastatic urothelial cancer, melanoma, and gastric cancer patients were relatively high (AUCs ranging from 0.67 to 0.88, Supplementary Table S4).

### Association between the TAE signature score and immunity in the CIT cohort

**FIGURE 3:**
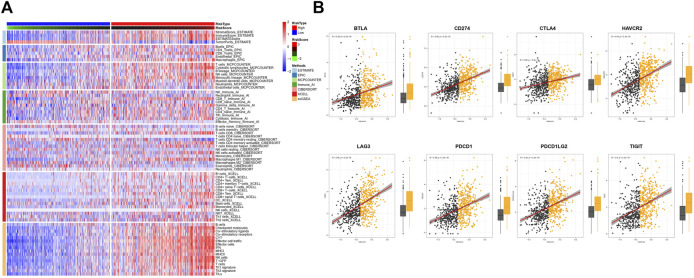
Tumor immunity analysis of the TAE score model. **(A)** Heatmap for immune responses based on ESTIMATE, EPIC, MCPcounter, Immune AI, CIBERSORT, xCell, and ssGSEA algorithms among high- and low-risk score groups. **(B)** Association between checkpoint molecules BTLA, CD274, CTLA4, HAVCR2, LAG3, PDCD1, PDCD1LG2, and BTLA, and TAE scores and their distribution in the low- and high score groups.

To uncover the immune activity of the TAE signature, ESTIMATE, EPIC, MCPcounter, Immune AI, CIBERSORT, xCell, and ssGSEA algorithms were used to estimate immune infiltration among high- and low score groups in all CIT subjects (Figure 3A). We observed that the tumor-killing immune cells, such as effector T cells, activated NK cells, M1 macrophage, and cytotoxic lymphocytes, were mainly distributed in high scores groups. In addition, the different expression levels of immune checkpoint genes between high- and low score groups were investigated. Patients with higher BTLA, CD274, CTLA4, HAVCR2, LAG3, PDCD1, PDCD1LG2, and BTLA presented a higher TAE score (Figure 3B).

### Pan-cancer profiling of TAE risk scores

**FIGURE 4:**
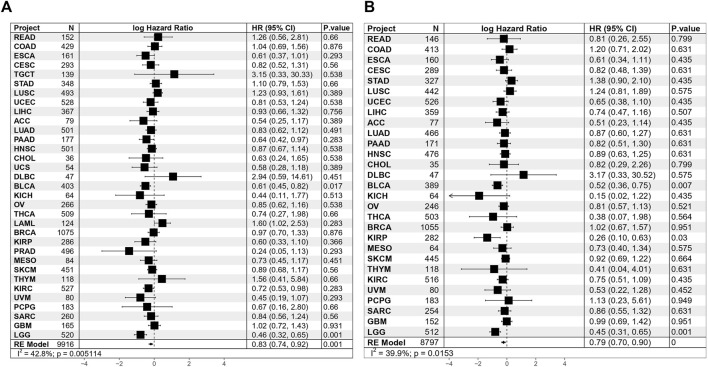
Results of Cox proportional hazards regression for OS **(A)** and DSS **(B)** analysis using TAE risk scores for 33 cancer types. Random effects meta-analysis was used to generate the pooled hazard ratios and *p*-values. The statistical test of heterogeneity is shown in the last column.

To explore the wider value of TAE scores in pan-cancers, data from 33 different cancer types in TCGA were used for further analysis. Univariate Cox regression analyses for the TCGA cohort suggested that the TAE score was associated with a good OS and disease-specific survival (DSS) in BLCA and LCG. In meta-analysis, the TAE score tends to be associated with a good prognosis in terms of OS and DSS (Figures 4A, B).

### TAE risk score significantly correlates with immunity in 33 cancer types

**FIGURE 5:**
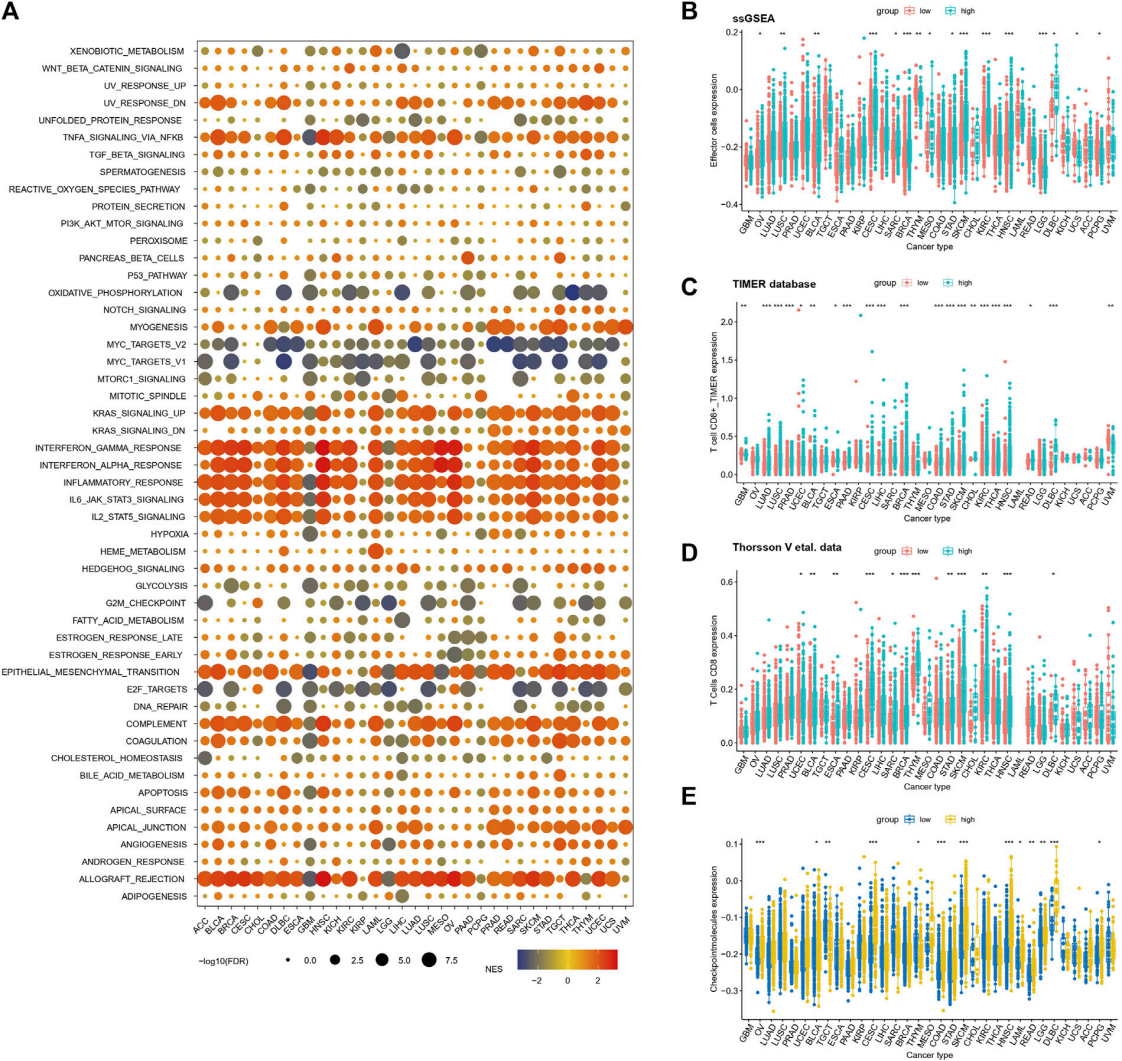
Relationships between inflammatory response risk scores and signaling pathways and immunophenotypes **(A)** Relationships between TAE risk scores and signaling pathways in cancer patients with high- and low-risk scores. Normalized enrichment scores and *p*-values were determined using the GSEA algorithm. **(B–D)** Infiltration levels of effector cells in the low- and high score groups were stratified by TAE risk scores in 33 cancer types from TCGA using ssGSEA analysis **(D,E)**, TIMER database **(C)**, and Thorsson et al.'s data **(D)** **(E)** Checkpoint molecule scores in low- and high-risk score groups stratified by the TAE scores in 33 cancer types.

In order to further elucidate the relationship between TAEs and cancer immunity across 33 cancer types, GSEA was performed, which suggested that gene sets involved in immune processes are consistently upregulated in the high score group, including interferon alpha/gamma response pathways and complement (Figure 5A). This result was further validated in Reactome gene sets (Supplementary Figure S3). Furthermore, we utilized marker gene expression analysis based on ssGSEA, the TIMER database, and Thorsson et al.’s data for illumination of the relationship between the risk score and effector cells. We observed that the consistent results from ssGSEA analysis (Figure 5B), TIMER database (Figure 5C), and Thorsson et al.'s data (Figure 5D) were that BLCA, CESC, BRCA, STAD, SKCM, KIRC, HNSC, and DLBCL had higher effector cell levels in high score groups than in low score groups (Mann–Whitney *U* test, *p* < 0.05). Subsequently, the estimation of the immune checkpoint levels revealed that 13 cancer types, namely, OV, BLCA, TGCT, CESC, THYM, COAD, SKCM, HNSC, LAML, READ, LGG, DLBCL, and PCPG, demonstrated a significant increase in checkpoint molecule scores in the high score group when compared to the low score group (Mann–Whitney *U* test, *p* < 0.05; Figure 5E).

## Discussion

The TECs are among the first cells to contact with immune cells while infiltrating from the circulation into the tumor tissue (Lambrechts et al., 2018). Tumor endothelial cells in the tumor microenvironment are important in cancer immunity, such as in the therapeutic response and in survival. In this study, we create a predictive model for determining the response to CIT therapy based on only six tumor endothelial gene markers. We observed that TAE scores can be a potential biomarker for the CIT response in multiple cancers. TECs are known to impact TME immunogenicity not only by actively guiding the circulating immune cells into the tumor stroma but also by fulfilling immune regulatory properties themselves, such as antigen presentation and T-cell priming functions (Georganaki et al., 2018; Nagl et al., 2020). The immunity analysis results in the CIT cohort revealed that tumor-killing immune cells, including effector T cells, activated NK cells, M1 macrophages, and cytotoxic lymphocytes, were more active in the high score group, which might explain why patients with high score were prone to have a better response to CIT and trend toward longer OS than low-risk score patients.

In order to further elucidate the role of TAE scores in clinical risk stratification, the association of TAEs and survival was assessed in 33 cancer types. Univariate Cox regression analysis showed the TAE risk score was significantly associated with OS and DSS in BLCA and LCG. Using a meta-analysis showed that high-risk scores were associated with a good prognosis, which may be ascribed to increased antitumor effectiveness related to the abundance of tumor-killing immune cells, particularly effector cells, which provide direct immune cytotoxicity.

TECs have shown to express known inhibitory immune checkpoint molecules, such as PD1, PDL1, and TIM3. PD-L2 can be upregulated through several pro-inflammatory cytokines including IFNγ and TNFα on TEC (Georganaki et al., 2018). The immune checkpoint molecule is currently the most frequently used biomarker in ICB treatment, guiding treatment decisions and patient stratification. In addition, the high score group had a higher expression trend of immune checkpoint associated genes in both the CIT cohort and in all 33 TCGA cancer types, indicating that patients in the high score group may have a better immunotherapy response.

In the TAE model, the risk score with the highest weight was assigned to *ADD1*, which contributed the most toward the model. ADD1 is known to promote the spectrin–actin assembly in erythrocytes and highly expressed in T cells (Robledo et al., 2008). The co-stimulation signal through CD28 was completely eliminated without ADD1, suggesting the role of actin-capping in T cells. ADD1 is also necessary for the complete activation of CD4^+^ T cells in response to antigens using a conditional knockout model (Thauland et al., 2019). In this study, the high score group with an immune activation environment enriched high levels of effector cells, probably due to the high expression levels of ADD1, in which CD28-mediated co-stimulation results in T-cell activation. The mechanism by which ADD1 facilitates co-stimulation is an important area for future studies to address. We hope that this article will promote future research in this important field of inquiry.

The present study has limitations. First, the datasets we were able to obtain were limited as we could only obtain over 700 patients with RNA-seq data and clinical response information. The extensive work of larger cohorts of immunotherapy is required to verify the TAE model in the future. Second, we lack clinical variable information in most cancer types in the CIT cohort, so we could not adjust their prognostic impact. Furthermore, patients from six different cancer types receiving different immunotherapy treatments may generate higher heterogeneity although we performed the sub-group analysis in some cancer types as well. Finally, the detailed mechanism on how each gene affects the response to immunotherapy is unclear, and it is nonetheless worthy of further study.

In summary, we developed a model using data from TAE gene expression which accurately predicts the response to CIT among different caner type patients. TAE score signatures derived from on-treatment samples have higher ability in predicting the efficacy of the CIT response in patients with metastatic melanoma. Our data also suggested that the TAE model is an independent prognostic factor used for predicting the response to CIT in metastatic urothelial cancer. Immunity analysis indicated that TAE is closely related to immune infiltration and immune checkpoint molecules. To the best of our knowledge, this is the first study to analyze TAEs in the CIT cohort and pan-cancer, highlighting the impact of TAEs on the immune response, potentially allowing more precise and personalized CIT in the future.

## Data Availability

The original contributions presented in the study are included in the article/Supplementary Material; further inquiries can be directed to the corresponding authors.

## Supplementary material

SUPPLEMENTARY FIGURE S1 Evaluation of the TAE risk score in tumor species of pre-treatment and on-treatment patients with metastatic melanoma. **(A,B)** Receiver operating characteristic curves of TAE scores in predicting immunotherapy responses between pre-treatment and on-treatment cohorts in patients with metastatic melanoma. **(C,D)** Kaplan–Meier curves of the TAE risk score. Kaplan–Meier curves for the overall survival of different TAE risk levels in the pre-treatment **(C)** and on-treatment cohorts **(D)**.

SUPPLEMENTARY FIGURE S2 Evaluation of the TAE risk score in metastatic urothelial cancer. **(A)** Receiver operating characteristic curves of TAE risk scores for predicting the immunotherapy response in patients with metastatic urothelial cancer. **(B)** Kaplan–Meier curves for the overall survival of different TAE risk levels in metastatic urothelial cancer cohorts. **(C)** Multi-variate regression analysis of overall survival in the metastatic urothelial cancer cohorts by using Cox regression analysis.

SUPPLEMENTARY FIGURE S3 Relationships between TAE risk scores and signaling pathways in cancer patients with high- and low-risk scores. Normalized enrichment scores and *p*-values were determined using the GSEA algorithm.
