# Supplementary figures and images for: A tumor-associated endothelial signature score model in immunotherapy and prognosis across pan-cancers
Source: Front Pharmacol. 2023 Aug 31;14:1190660. doi: 10.3389/fphar.2023.1190660 (PMC10500301; doi:10.3389/fphar.2023.1190660)

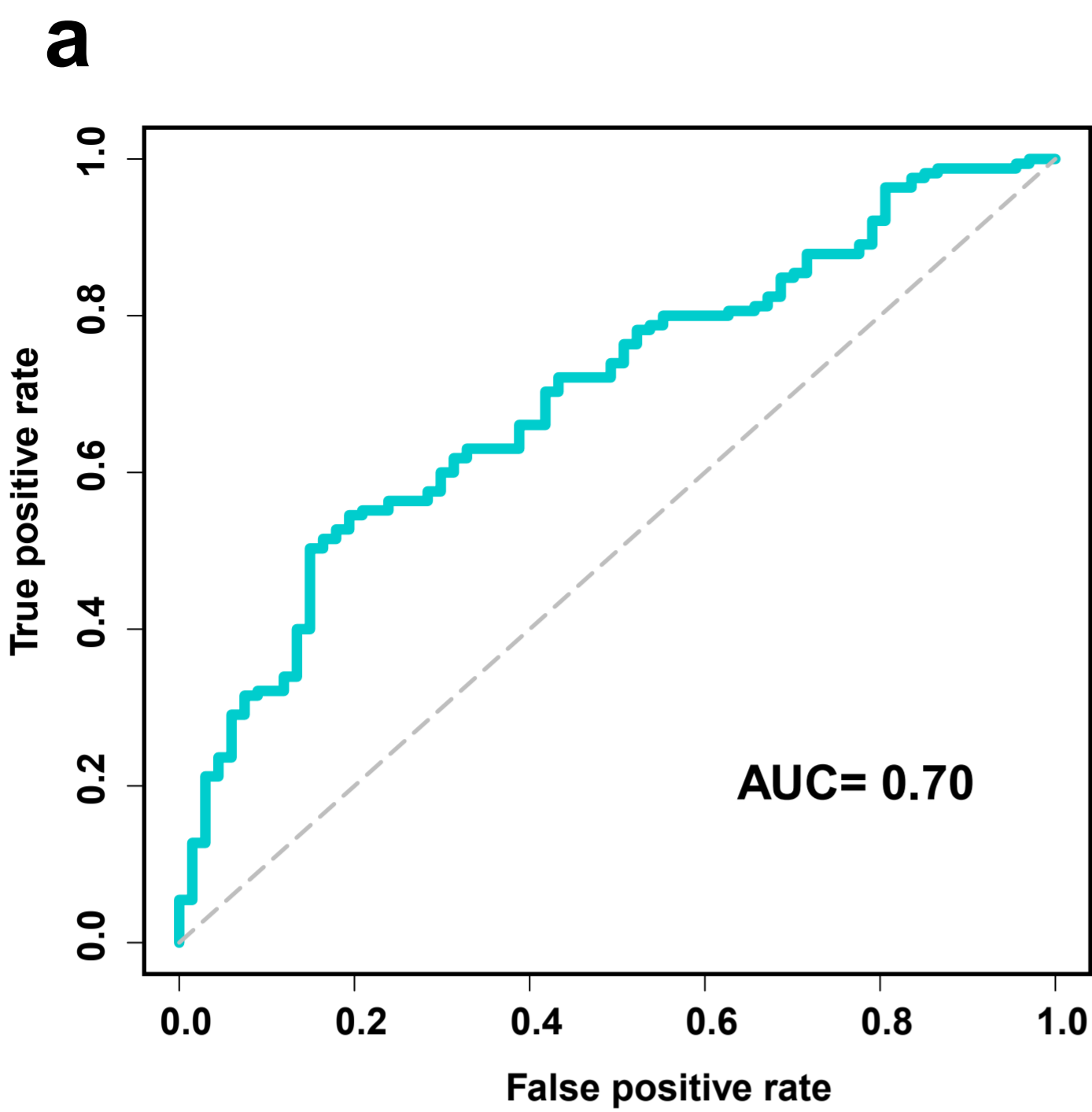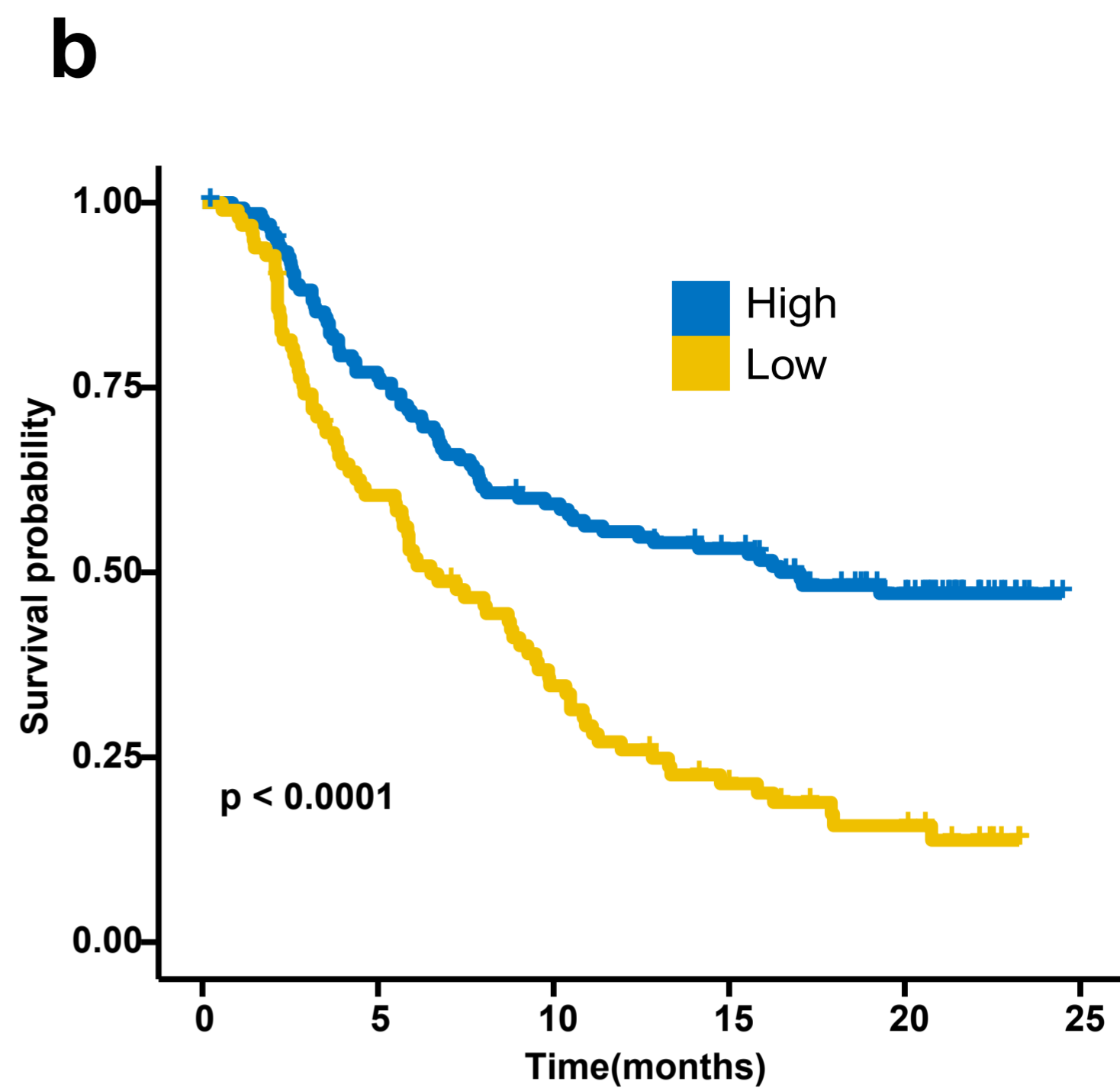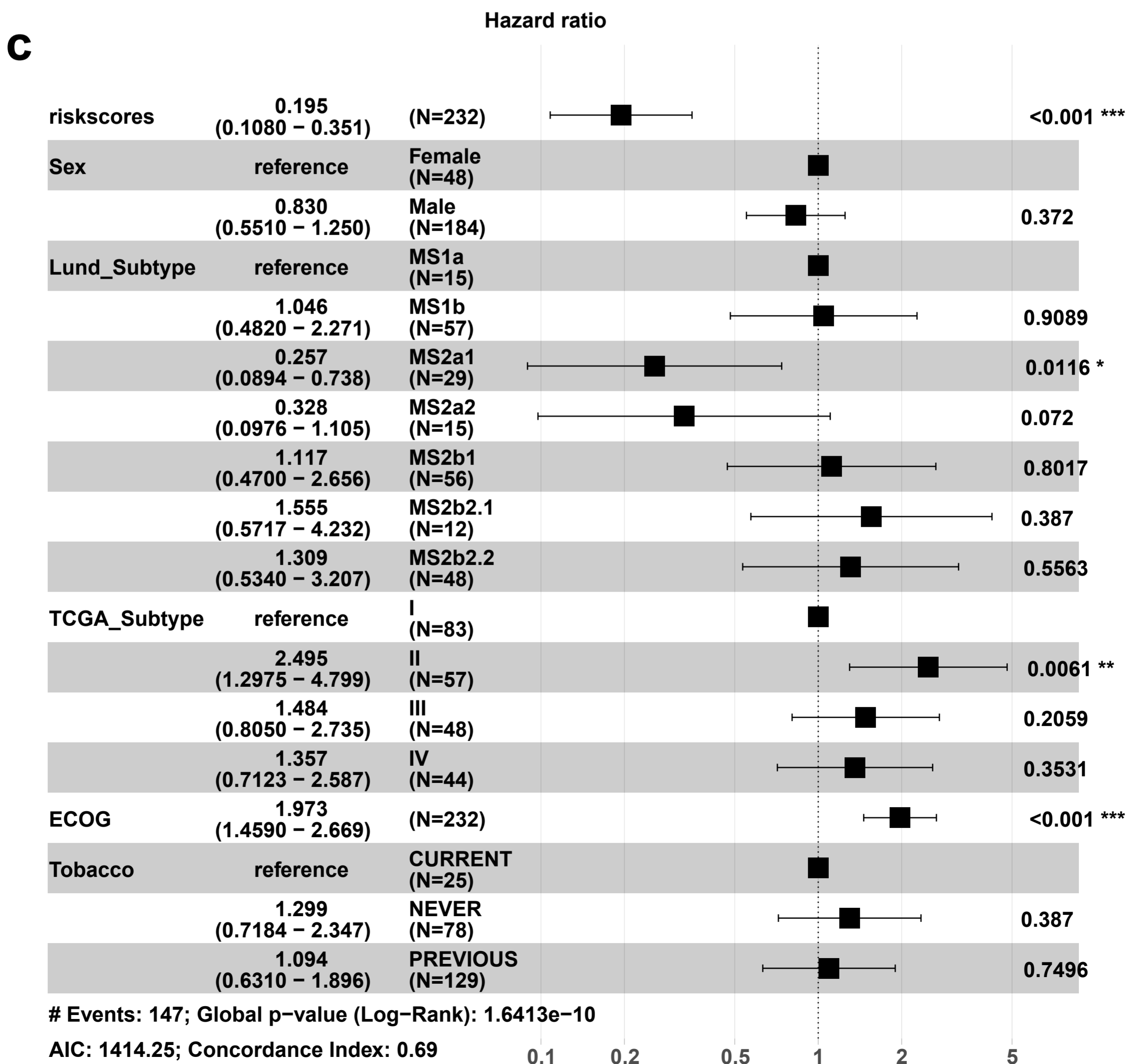

Supplement: Supplementary file 1 [file DataSheet2.PDF]

**a****pre-treatment cohort(n=264)**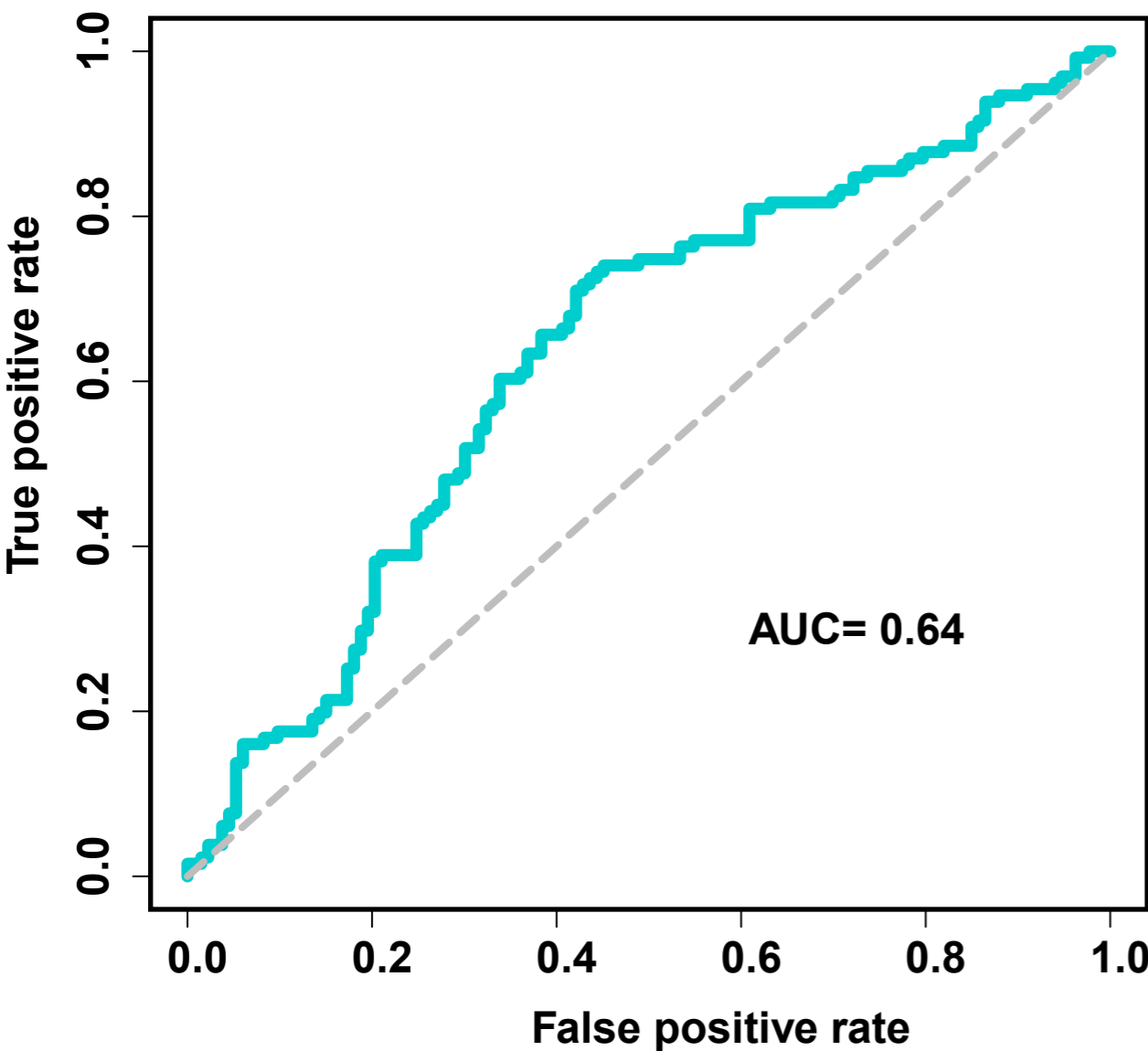**b****on-treatment cohort(n=84)**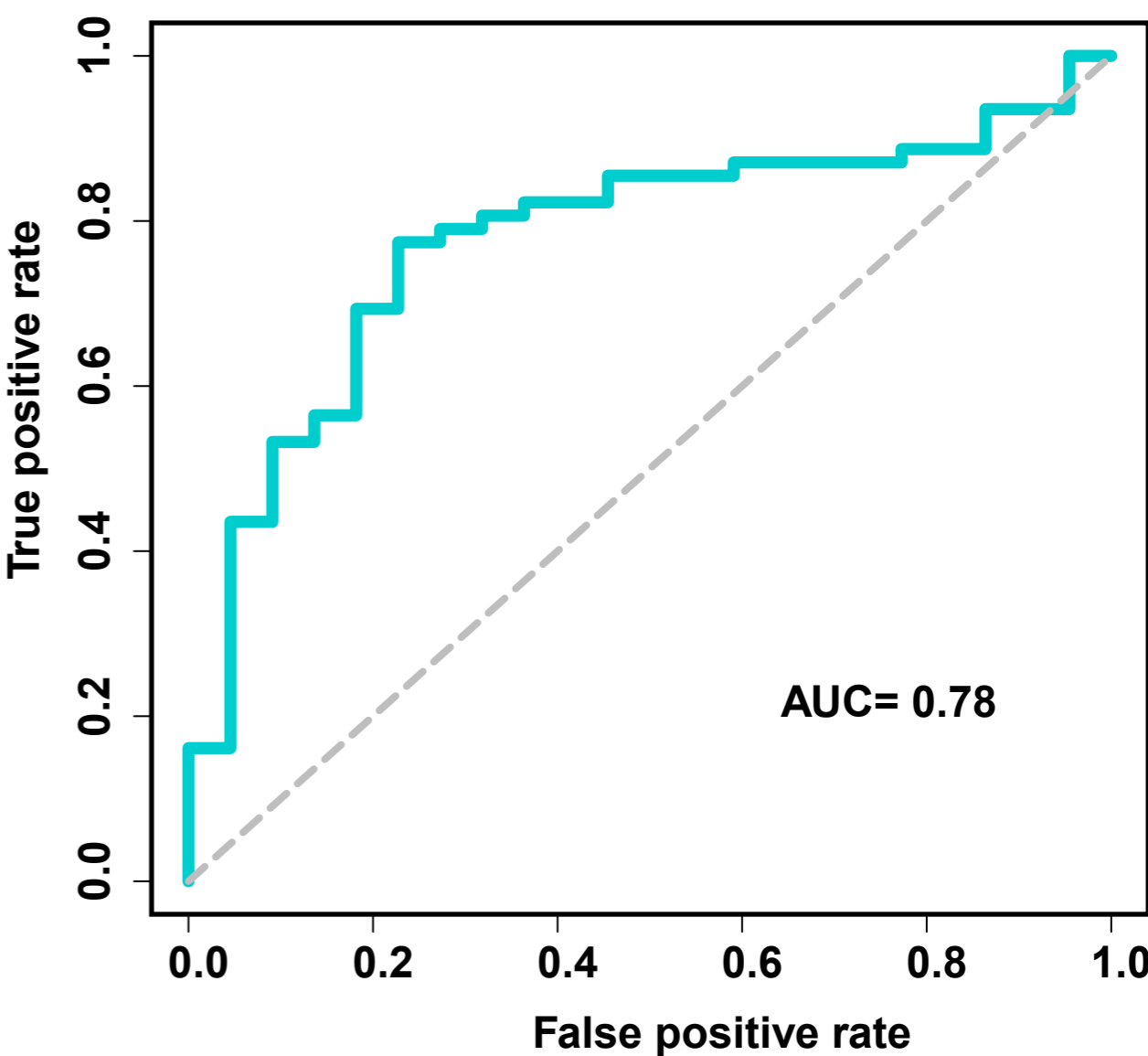**c**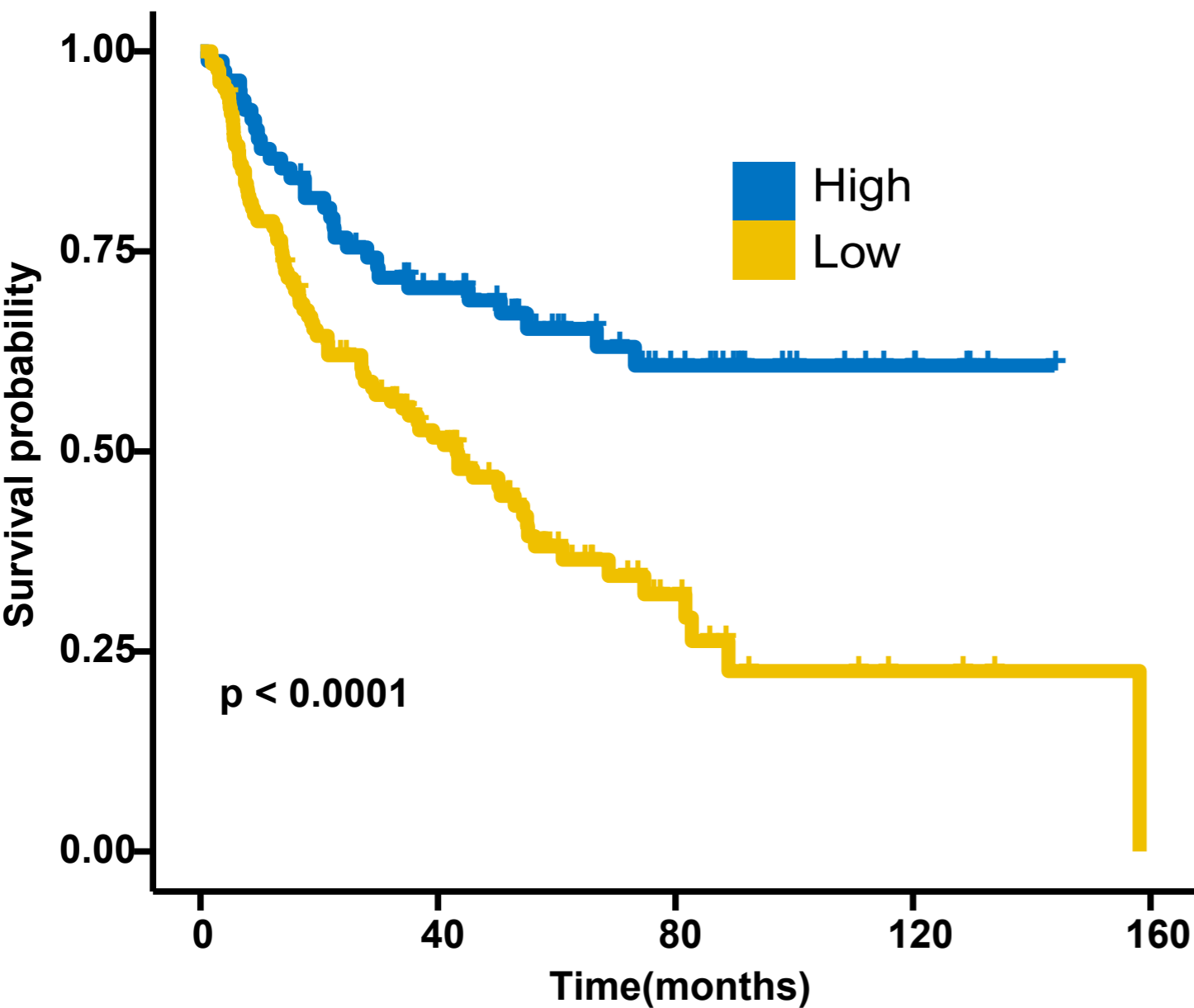**d**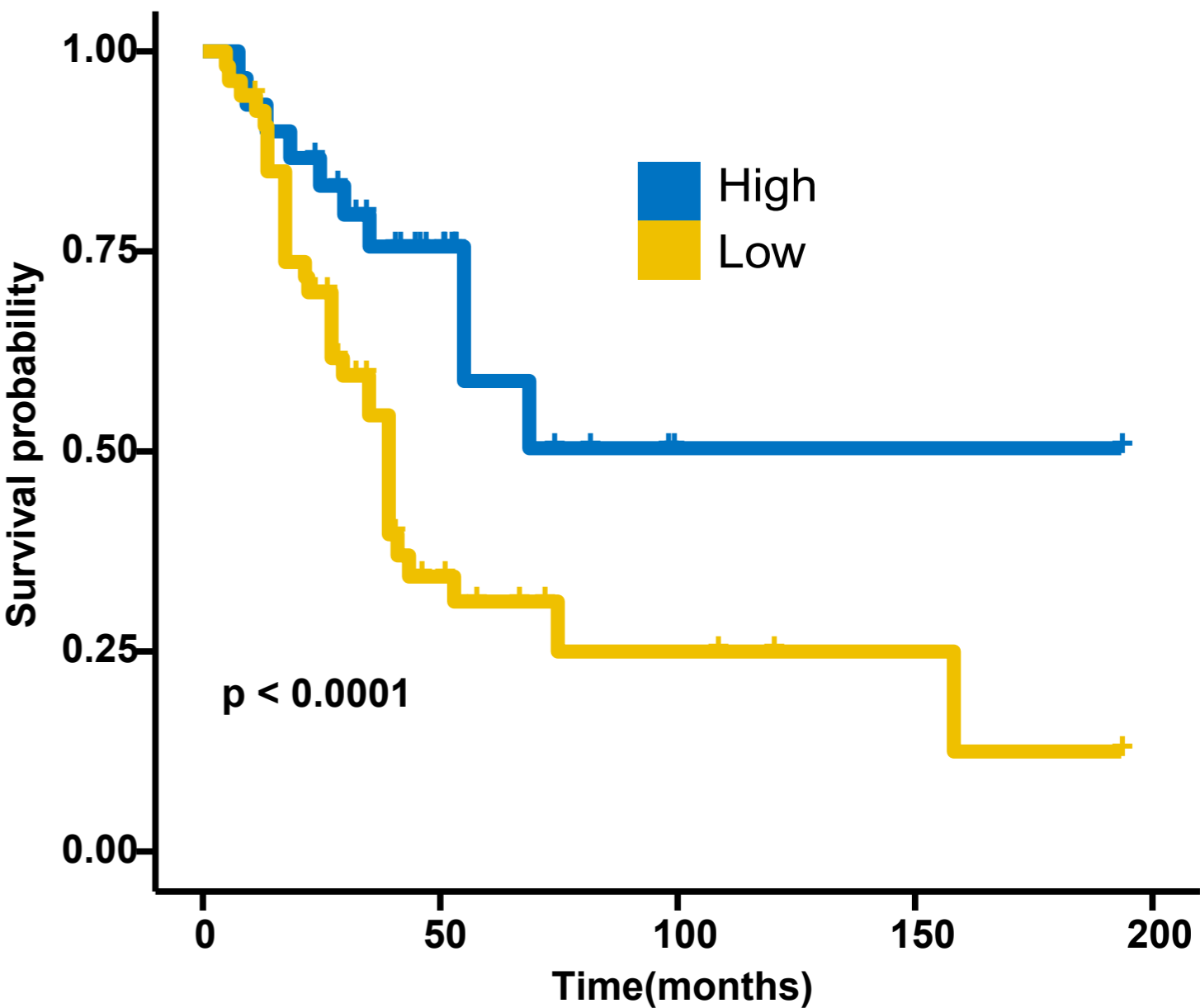

Supplement: Supplementary file 3 [file DataSheet1.PDF]
