# Supplementary material for: A tumor-associated endothelial signature score model in immunotherapy and prognosis across pan-cancers
Source: Front Pharmacol. 2023 Aug 31;14:1190660. doi: 10.3389/fphar.2023.1190660 (PMC10500301; doi:10.3389/fphar.2023.1190660)

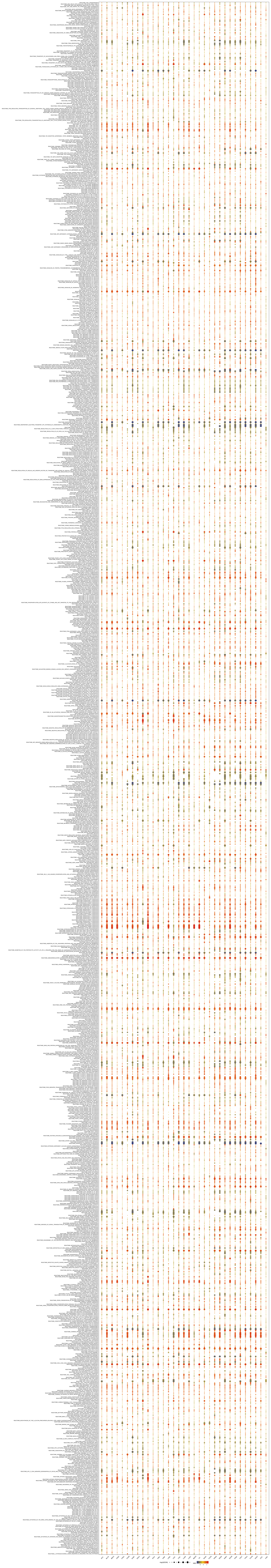

Supplement: Supplementary file 2 [file DataSheet3.PDF]
